# Eating‐ and oral health‐related quality of life in patients under fixed orthodontic treatment

**DOI:** 10.1002/cre2.631

**Published:** 2022-07-09

**Authors:** Yasamin Babaee Hemmati, Arastoo Mirmoayed, Mohammad Ebrahim Ghaffari, Mehran Falahchai

**Affiliations:** ^1^ Department of Orthodontics, School of Dentistry, Dental Sciences Research Center Guilan University of Medical Sciences Rasht Iran; ^2^ School of Dentistry, Dental Sciences Research Center Guilan University of Medical Sciences Rasht Iran; ^3^ Department of Prosthodontics, School of Dentistry, Dental Sciences Research Center Guilan University of Medical Sciences Rasht Iran

**Keywords:** eating, eating behavior, orthodontic treatment, quality of life

## Abstract

**Objective:**

The eating problems and changes in the diet and dietary intake of patients under orthodontic treatment are of great importance, and the available studies on this topic are mostly qualitative. Thus, this study aimed to assess the oral health‐related quality of life (OHRQoL) and the eating‐related quality of life (ERQoL) of patients under fixed orthodontic treatment.

**Materials and Methods:**

This prospective study evaluated 105 patients (65 males, 40 females) with a mean age of 26 ± 1.1 years, who required fixed orthodontic treatment. All participants filled out the Oral Health Impact Profile‐14 (OHIP‐14) questionnaire before treatment (T0), and at 1 (T1), 3 (T2), and 6 (T3) months after treatment, and the ERQoL questionnaire at 1 (T1), 3 (T2), and 6 (T3) months after treatment. Data were analyzed using repeated measures analysis of variance with Greenhouse–Geisser and Huynh‐Feldt corrections and Bonferroni test for pairwise comparisons (*α* = .05).

**Results:**

The OHIP‐14 total score increased at T1 and decreased at T2 and T3, with significant differences between all four time points (*p* < .001). The ERQoL total score decreased over time, and significant differences were noted between all three time points (*p* < .001). No significant difference existed in OHRQoL or ERQoL with regard to gender at any time point (*p* > .05).

**Conclusion:**

The reduction in OHRQoL due to fixed orthodontic treatment was temporary and improved over time. The eating problems also decreased with time.

## 1 BACKGROUND

Malocclusion is among the most common dental anomalies in most countries worldwide (Gatto et al., 2019; Jena et al., 2020; Sobouti et al., 2020). Malocclusion refers to a noticeable change in normal dental occlusion, and is considered as a complex of deviations from the normal state rather than a disease (Agbaje et al., 2018; Choi et al., 2017). It can lead to problems such as dissatisfaction with the appearance, impaired mastication, suboptimal quality of the function of the temporomandibular joints, difficult deglutition, speech impairment, higher susceptibility to traumatic dental injuries, periodontal disease, and dental caries (Navabi et al., 2012; Thomson & Broder, 2018). Thus, the demand for fixed orthodontic treatment has recently increased, particularly among the adult population, to overcome such problems (de Couto Nascimento et al., 2016; Gao et al., 2021; Johal et al., 2015). Elimination of psychosocial problems has been mentioned as the main reason behind the increased demand for orthodontic treatment (Gatto et al., 2019).

In the past, most clinicians used tools based on clinical results to interpret the orthodontic treatment outcome. However, in the recent years, researchers are more interested in using tools measuring the oral health status based on the data collected from patients under treatment. By doing so, they can obtain a more accurate understanding of the needs and satisfaction rate of patients with regard to the use of fixed orthodontic appliances (Demirovic et al., 2019). The concept of oral health‐related quality of life (OHRQoL) indicates the health status of the oral cavity, dentition, and related tissues, enabling eating, speech, and socialization with no problem or concern (Johal et al., 2015). In fact, this index not only evaluates the oral signs and symptoms and the related physical limitations, but also analyzes their effects on the psychosocial condition of individuals (Sun et al., 2018). Evidence shows that orthodontic treatment can have different physical, psychological, and social effects on the QoL of orthodontic patients (Abreu et al., 2013; Jena et al., 2020). Thus, the concept of OHRQoL can be used as a suitable index for quantification of the orthodontic treatment results, and increase the motivation of patients to continue their orthodontic treatment (Jena et al., 2020; Johal et al., 2015). Depending on the orthodontic treatment phase, OHRQoL may improve or deteriorate (Chen et al., 2010; Jena et al., 2020; Liu et al., 2011; Zhang et al., 2008).

The majority of patients under fixed orthodontic treatment experience moderate to severe problems in biting and chewing of hard foods. These problems lead to extensive changes in the diet and eating behavior of patients (Trein et al., 2013). Ostasevic et al. (2006) found that problems related to biting and chewing foods were the most commonly reported problems by the adolescents under fixed orthodontic treatment, and such problems were reported by half of the participants. On the other hand, pain during orthodontic treatment is reported by the majority of patients, and some authors have reported a significant correlation between pain and eating behavior of patients (Abed Al Jawad et al., 2012). Nonetheless, most studies only used general questionnaires with limited questions regarding eating problems in one or two time intervals, for example, before or after orthodontic treatment, and therefore, could not offer a clear picture of the problem. Resultantly, many eating‐related problems in orthodontic patients such as the pleasure of eating, changes in diet, and eating‐related emotions and behaviors have not been precisely addressed.

Studies focusing on the dietary changes and eating problems of patients under orthodontic treatment are mostly qualitative (Abed Al Jawad et al., 2012; Choi et al., 2017), and no quantitative study has addressed these topics in different time periods during the course of orthodontic treatment. Thus, further investigations are required on this topic to find a solution and minimize the related physical and psychological problems in orthodontic patients. Also, the patients should be empowered to accept and cope with the problems related to fixed orthodontic treatment. This study aimed to assess the OHRQoL and eating‐related quality of life (ERQoL) of patients under fixed orthodontic treatment. The null hypothesis was that the OHRQoL and ERQoL would not be influenced by fixed orthodontic treatment at any of the assessed time points in this study, and gender would have no significant effect on OHRQoL or ERQoL either.

## 2 MATERIALS AND METHODS

This study was approved by the ethics committee of our university, and evaluated patients presenting to the dental school clinic and two private offices. The sample size was calculated to be 94 assuming 80% study power using the formula below:

$$\begin{array}{l} n_{0}=\frac{2{\left((,z_{\frac{\alpha }{2}}+z_{\beta }\right)}^{2}\sigma^{2}\left((,1+\left((,m-1,)\right)\rho ,)\right)}{md^{2}}=\frac{2{\left((,1.96+0.84,)\right)}^{2}{\left((,10.2,)\right)}^{2}\left((,1+\left((,3,)\right)0.5,)\right)}{4{\left((,3.3,)\right)}^{2}}=93.63≃94,\\ n=n_{0}\times \frac{1}{1-f}=104.44≅105, \end{array}$$

where *Z*_*α*/2_ = 1.96, *σ* = 10.2 (standard deviation), *m* = 4 (replications), *d* = 3.3 (effect size), *ρ* = 0.50 (correlation), 1 − *β* = 80% (statistical power), and *α* = .05 (error level). Considering the possibility of 10% dropouts, the final sample size was increased to 105. Accordingly, 105 healthy individuals (65 males and 40 females) with a mean age of 26 ± 1.1 years participated in this prospective study.

The inclusion criteria were requiring fixed orthodontic treatment of both the maxilla and mandible, Angle class I malocclusion, having a complete set of erupted teeth, dental crowding manageable by nonextraction orthodontic treatment, willingness for participation in the study, and regular attendance in the monthly follow‐ups.

The exclusion criteria were history of medical and cognitive problems, history of maxillofacial surgery or orthodontic treatment, developmental anomalies such as the cleft lip and/or palate and craniofacial anomalies, significant temporomandibular disorders, requiring two‐stage orthodontic treatment (one stage during the growth period followed by fixed orthodontic treatment), poor periodontal health, untreated dental caries, presence of impacted or semi‐impacted teeth, presence of conditions affecting the diet or following a particular diet, poor cooperation, and not showing up for the monthly follow‐ups. All patients signed informed consent forms before participation in the study, and received adequate instructions on how to fill out the questionnaires.

The Dental Health Component of the Index of Orthodontic Treatment Need (IOTN‐DHC) was used to assess the severity of malocclusion and the orthodontic treatment need of patients (Palomares et al., 2012). Accordingly, the patients were categorized into five grades: Grade I: no need for treatment, Grade II: little need for treatment, Grade III: moderate or borderline need for treatment, Grade IV: great need for treatment, and Grade V: very great need for treatment (Palomares et al., 2012). This classification is based on the following 10 items: overjet, reverse overjet, overbite, open bite, cross bite, crowding, impeded eruption, cleft lip and palate or other craniofacial anomalies, class II and III buccal occlusion, and hypodontia (Choi et al., 2016).

To assess the OHRQoL, the Persian version of the Oral Health Impact Profile‐14 (OHIP‐14) was used. The validity and reliability of the Persian version of the OHIP‐14 have been previously confirmed (Asgari et al., 2013; Motallebnejad et al., 2011; Navabi et al., 2012). This questionnaire was administered among patients at four time points namely before the treatment onset (T0), at 1 month after the onset of treatment (T1), at 3 months after the onset of treatment (T2), and at 6 months after the onset of treatment (T3). This questionnaire includes 14 questions in seven domains of functional limitations, physical pain, psychological discomfort, physical disability, psychological disability, social disability, and handicap. The scoring system was based on the Likert's five‐point scale as follows: 0: never, 1: rarely, 2: occasionally, 3: most of the time, and 4: almost always. The OHIP‐14 total score for each patient was the sum of individual scores of 14 questions, and ranged from 0 to 56. Higher scores indicated poorer OHRQoL.

The ERQoL was also assessed in orthodontic patients. For this purpose, the questionnaire used by Abdulrahman et al. (2018) was translated to Persian using the standard forward–backward translation method. The original version of the questionnaire was first translated from English to Persian by three translators independently. The translated versions were then analyzed to obtain one single version. This version was then back‐translated to English by three other translators independently who did not have access to the original English version of the questionnaire. Finally, the back‐translated version and the original English version of the questionnaire were compared. Since some of the questions were not suitable for comparison of several time intervals during the course of treatment, the necessary modifications were made, and the final version of the questionnaire was designed. To quantitatively assess the content validity of the questionnaire, the content validity ratio (CVR) and the content validity index (CVI) were calculated. For this purpose, 10 orthodontists evaluated the questions one by one. The questions that did not acquire the minimum required score were excluded. The primary version of the questionnaire had 28 questions in six domains, which decreased to 17 questions in six domains due to inadequate content validity. After excluding the unsuitable questions, the CVI was calculated to be 0.99, and the CVR was calculated to be 0.95. The Cronbach's *α* was calculated to be .93. The scoring system of this questionnaire was based on the visual analog scale. The score of each question ranged from 1 to 10 with 10 indicating the worst and 1 indicating the best situation. Thus, the total score ranged from 17 to 170; higher scores indicated poorer ERQoL. This questionnaire was administered among patients at three time points namely at 1 month after treatment (T1), at 3 months after treatment (T3), and at 6 months after treatment (T3).

For the purpose of standardization, the examiners were calibrated with regard to the therapeutic and nutritional instructions and protocols, and all procedures were conducted under the supervision of the authors. Accordingly, the private offices of the examiners who were the university faculty members and practiced in the dental school clinic were chosen for patient selection to enhance coordination.

The normality of data distribution was evaluated by the Shapiro–Wilk and kurtosis and skewness tests. The homogeneity of variances was assessed by the Levene's test. Data were analyzed using repeated measures analysis of variance and Greenhouse–Geisser and Huynh‐Feldt corrections, if required. The Bonferroni test was applied for pairwise comparisons of the time points. All statistical analyses were carried out using SPSS version 24 at .05 level of significance.

## 3 RESULTS

Of 105 orthodontic patients participating in this study, 61.9% (*n* = 65) were males and 38.1% (*n* = 40) were females. The participants were between 12 and 40 years with a mean age of 26 ± 1.1 years. Of 105 participants, 14 had ceramic and 91 had metal brackets. Also, according to the IOTN‐DHC classification, 86 patients (81.9%) were Grade III (moderate need for treatment), 11 (10.5%) were Grade II (little need for treatment), and 8 (7.6%) were Grade IV (great need for treatment). Moreover, 100 patients (95%) were recruited from the dental school clinic and the remaining were recruited from the private offices.

Table 1 presents the results regarding the OHRQoL of patients according to the OHIP‐14 questionnaire domains and questions. According to the results of repeated measures analysis of variance with Greenhouse–Geisser correction and pairwise comparisons by the Bonferroni test, the trend of change in OHIP‐14 total score was descending over time (except for the T0–T1 time interval), which indicates an improvement in OHRQoL. Pairwise comparisons of the time points revealed significant differences between all time points (*p* < .001, Table 2).

**Table 1:** OHRQoL scores of patients at different time point

Items	OHIP dimensions	T0 (mean ± SD)	T1 (mean ± SD)	T2 (mean ± SD)	T3 (mean ± SD)	*p* Value (F)
Domain 1: Functional limitations						
1	Had trouble in pronouncing words?	1.68 ± 0.54	1.72 ± 0.70	1.96 ± 0.84	1.51 ± 0.76	<.001 (7.94)
2	Felt that sense of taste had worsened?	1.64 ± 0.53	1.93 ± 0.71	1.75 ± 0.43	1.32 ± 0.61	<.001 (20.68)
Total (1 ± 2)		3.31 ± 0.76	3.65 ± 1.29	3.71 ± 0.90	2.83 ± 0.96	<.001 (17.95)
Domain 2: Physical pain						
3	Had painful aching in your mouth?	1.54 ± 0.50	2.16 ± 0.74	1.09 ± 0.50	0.85 ± 0.51	<.001 (119.18)
4	Uncomfortable when eating food?	1.53 ± 0.52	2.16 ± 0.84	1.52 ± 0.55	0.94 ± 0.69	<.001 (62.62)
Total (3 ± 4)		3.07 ± 0.71	4.32 ± 1.36	2.60 ± 0.75	1.79 ± 0.88	<.001 (138.83)
Domain 3: Psychological discomfort						
5	Has been feeling self‐conscious?	1.47 ± 0.50	2.11 ± 0.81	0.56 ± 0.60	0.48 ± 0.53	<.001 (221.75)
6	Had felt tense?	1.69 ± 0.56	2.09 ± 0.80	1.96 ± 0.72	1.82 ± 0.83	<.002 (6.86)
Total (5 ± 6)		3.15 ± 0.78	4.20 ± 1.34	2.52 ± 0.85	2.29 ± 0.93	<.001 (89.83)
Domain 4: Physical disability						
7	Diet has been unsatisfactory?	1.58 ± 0.53	2.01 ± 0.89	1.37 ± 0.52	1.24 ± 0.59	<.001 (29.35)
8	Has had to interrupt meals?	1.49 ± 0.50	1.98 ± 0.66	1.76 ± 0.51	1.65 ± 0.57	<.001 (16.62)
Total (7 ± 8)		3.06 ± 0.66	4.00 ± 1.29	3.13 ± 0.77	2.88 ± 0.91	<.001 (32.64)
Domain 5: Psychological disability						
9	Finds it difficult to relax?	1.46 ± 0.50	2.14 ± 0.72	1.44 ± 0.49	1.38 ± 0.50	<.001 (47.95)
10	Has been a bit embarrassed?	1.62 ± 0.52	2.18 ± 0.94	1.50 ± 0.62	1.39 ± 0.61	<.001 (29.74)
Total (9 ± 10)		3.07 ± 0.71	4.32 ± 1.52	2.94 ± 0.76	2.77 ± 0.81	<.001 (57.42)
Domain 6: Social disability						
11	Has been irritable with other people?	1.90 ± 0.53	2.14 ± 0.90	1.55 ± 0.51	1.42 ± 0.61	<.001 (31.59)
12	Has had difficulty during usual jobs?	1.78 ± 0.57	2.18 ± 0.84	1.41 ± 0.51	1.31 ± 0.54	<.001 (43.14)
Total (11 ± 12)		3.67 ± 0.81	4.32 ± 1.60	2.96 ± 0.71	2.73 ± 0.81	<.001 (54.07)
Domain 7: Handicap						
13	Life has been less satisfying?	2.09 ± 0.53	2.04 ± 0.90	1.75 ± 0.43	1.61 ± 0.52	<.001 (15.50)
14	Has been totally unable to function?	1.65 ± 0.48	1.87 ± 0.84	1.06 ± 0.51	1.04 ± 0.51	<.001 (59.37)
Total (13 ± 14)		3.73 ± 0.66	3.92 ± 1.56	2.80 ± 0.66	2.64 ± 0.79	<.001 (49.12)
Total OHIP‐14 score		23.09 ± 1.88	28.76 ± 7.63	20.69 ± 2.16	17.96 ± 2.63	<.001 (124.87)

*Note*: An SD indicates standard deviation; T0, baseline data; T1, 1 month after the onset of orthodontic treatment; T2, 3 months after the onset of orthodontic treatment; T3, 6 months after the onset of orthodontic treatment.

Abbreviations: NS, nonsignificant; OHIP‐14, Oral Health Impact Profile 14; OHRQoL, oral health‐related quality of life.

**Table 2:** Pairwise comparisons of OHRQoL scores at different time points

Items	OHIP dimensions	T0–T1	T0–T2	T0–T3	T1–T2	T1–T3	T2–T3
Domain 1: Functional limitations							
1	Had trouble in pronouncing words?	*	NS	NS	NS	NS	***
2	Felt that sense of taste had worsened?	**	NS	***	NS	***	***
Total (1 ± 2)		NS	**	***	NS	***	***
Domain 2: Physical pain							
3	Had painful aching in your mouth?	***	***	***	***	***	***
4	Uncomfortable when eating food?	***	NS	***	***	***	***
Total (3 ± 4)		***	***	***	***	***	***
Domain 3: Psychological discomfort							
5	Has been feeling self‐conscious?	***	***	***	***	***	*
6	Had felt tense?	***	***	NS	NS	NS	*
Total (5 ± 6)		***	***	***	***	***	***
Domain 4: Physical disability							
7	Diet has been unsatisfactory?	***	NS	NS	***	***	*
8	Has had to interrupt meals?	***	***	NS	*	***	*
Total (7 ± 8)		***	NS	NS	***	NS	**
Domain 5: Psychological disability							
9	Finds it difficult to relax?	***	NS	NS	***	***	NS
10	Has been a bit embarrassed?	***	NS	*	***	***	**
Total (9 ± 10)		***	NS	*	***	***	***
Domain 6: Social disability							
11	Has been irritable with other people?	NS	***	***	***	***	*
12	Has had difficulty during usual jobs?	***	***	***	***	***	**
Total (11 ± 12)		**	***	***	***	***	***
Domain 7: Handicap							
13	Life has been less satisfying?	NS	***	***	*	***	**
14	Has been totally unable to function?	NS	***	***	***	***	NS
Total (13 ± 14)		NS	***	***	***	***	**
Total OHIP‐14 score		***	***	***	***	***	***

*Note*: An SD indicates standard deviation; T0, baseline data; T1, 1 month after the onset of orthodontic treatment; T2, 3 months after the onset of orthodontic treatment; T3, 6 months after the onset of orthodontic treatment.

Abbreviations: NS, nonsignificant; OHIP‐14, Oral Health Impact Profile 14; OHRQoL, oral health‐related quality of life.

* *p* < .05;

** *p* < .01;

*** *p* < .001.

Table 3 presents the details regarding the ERQoL questionnaire including the domain and question scores at different time points. The results of repeated measures analysis of variance with Greenhouse–Geisser correction and pairwise comparisons by the Bonferroni test revealed that the trend of change in the total score of ERQoL was descending and significant (*p* < .001). Pairwise comparisons of the time points showed significant differences between all time points (*p* < .001, Table 4).

**Table 3:** ERQoL scores of patients at different time points

Items	ERQoL dimensions	T1 (mean ± SD)	T2 (mean ± SD)	T3 (mean ± SD)	*p* Value (F)
Domain 1: From the start and afterwards					
1	How do you think eating with a brace will be?	7.64 ± 1.79	7.45 ± 0.98	7.43 ± 0.96	.309 (1.18)
2	On the first day after your usual visits for tightening the brace what happened to your eating?	7.80 ± 1.67	7.19 ± 0.87	7.18 ± 0.77	.002 (10.11)
3	How did you find eating over time at time intervals between your visits?	6.60 ± 1.91	6.87 ± 0.55	5.82 ± 0.85	<.001 (18.86)
Total (1 ± 2 ± 3)		22.05 ± 4.02	21.40 ± 1.37	20.44 ± 1.59	.001 (10.79)
Domain 2: Eating with your brace					
4	When eating with your brace, how do you find biting foods?	7.76 ± 1.88	6.87 ± 0.82	6.10 ± 0.96	<.001 (45.60)
5	When eating with your brace, how do you find chewing foods?	7.78 ± 1.76	7.01 ± 0.88	6.04 ± 0.82	<.001 (56.95)
Total (4 ± 5)		15.54 ± 3.40	13.87 ± 1.26	12.14 ± 1.24	<.001 (67.36)
Domain 3: Changes that happened					
6	With your brace, how much food do you eat compared with before you had your brace?	6.54 ± 1.71	6.35 ± 0.58	5.59 ± 0.67	<.001 (20.64)
7	How long does it take you to eat with your brace compared to when you did not have it?	6.73 ± 1.73	6.63 ± 0.65	5.61 ± 0.71	<.001 (32.32)
8	Since wearing your brace, how does your food taste?	5.26 ± 1.29	4.93 ± 0.50	4.91 ± 0.52	.012 (6.41)
Total (6 ± 7 ± 8)		18.54 ± 3.67	17.91 ± 0.95	16.11 ± 1.10	<.001 (32.52)
Domain 4: Surrounding people and venue of eating					
9	How do you feel when eating with your brace in front of your family?	4.72 ± 2.10	1.71 ± 0.66	1.57 ± 0.58	<.001 (186.16)
10	How do you feel when eating with your brace in front of your friends?	4.72 ± 2.19	1.58 ± 0.55	1.48 ± 0.53	<.001 (193.33)
11	How do you feel when eating with your brace in presence of people you don't know?	4.86 ± 2.18	2.59 ± 0.81	2.14 ± 0.64	<.001 (121.13)
12	Since wearing your brace do you accept invitations to meals or parties?	4.72 ± 1.20	4.86 ± 0.56	4.90 ± 0.43	.246 (1.37)
Total (9 ± 10 ± 11 ± 12)		19.03 ± 6.63	10.74 ± 1.22	10.08 ± 1.11	<.001 (161.89)
Domain 5: You and your dentist					
13	How helpful did you find the instructions your dentist gave you about eating with your brace?	2.98 ± 2.06	1.93 ± 0.76	1.84 ± 0.66	<.001 (27.03)
14	Did the advice of your dentist make you change the foods you eat?	3.75 ± 1.99	1.57 ± 0.60	1.52 ± 0.53	<.001 (122.23)
15	How often do you avoid eating foods if you are unable to brush your teeth/clean brace after meal?	3.30 ± 1.75	1.39 ± 0.50	1.42 ± 0.51	<.001 (111.41)
Total (13 ± 14 ± 15)		10.03 ± 3.96	4.89 ± 1.10	4.78 ± 0.98	<.001 (176.26)
Domain 6: Enjoyment of food					
16	When wearing your brace, can you eat the foods you want to?	6.15 ± 2.01	2.52 ± 0.87	2.03 ± 0.79	<.001 (306.37)
17	Do you feel embarrassed when you eat with your brace?	2.18	5.50 ± 0.72	5.27 ± 0.88	<.001 (30.64)
Total (16 ± 17)		10.21 ± 2.38	8.01 ± 1.06	7.29 ± 1.20	<.001 (105.71)
Total score		95.43 ± 15.18	76.85 ± 3.18	70.86 ± 3.30	<.001 (206.65)

*Note*: An SD indicates standard deviation; T0, baseline data; T1, 1 month after the onset of orthodontic treatment; T2, 3 months after the onset of orthodontic treatment; T3, 6 months after the onset of orthodontic treatment.

Abbreviations: NS, nonsignificant; OHIP‐14, Oral Health Impact Profile 14; ERQoL, eating‐related quality of life.

**Table 4:** Pairwise comparisons of ERQoL scores at different time points

Items	ERQoL dimensions	T1–T2	T1–T3	T2–T3
Domain 1: From the start and afterwards				
1	How do you think eating with a brace will be?	NS	NS	NS
2	On the first day after your usual visits for tightening the brace what happened to your eating?	**	**	NS
3	How did you find eating over time at time intervals between your visits?	NS	***	NS
Total (1 ± 2 ± 3)		NS	***	***
Domain 2: Eating with your brace				
4	When eating with your brace, how do you find biting foods?	***	***	***
5	When eating with your brace, how do you find chewing foods?	***	***	***
Total (4 ± 5)		***	***	***
Domain 3: Changes that happened				
6	With your brace, how much food do you eat compared with before you had your brace?	NS	***	***
7	How long does it take you to eat with your brace compared to when you did not have it?	NS	***	***
8	Since wearing your brace, how does your food taste?	*	*	NS
Total (6 ± 7 ± 8)		NS	***	***
Domain 4: Surrounding people and venue of eating				
9	How do you feel when eating with your brace in front of your family?	***	***	***
10	How do you feel when eating with your brace in front of your friends?	***	***	**
11	How do you feel when eating with your brace in presence of people you don't know?	***	***	***
12	Since wearing your brace do you accept invitations to meals or parties?	NS	NS	NS
Total (9 ± 10 ± 11 ± 12)		***	***	***
Domain 5: You and your dentist				
13	How helpful did you find the instructions your dentist gave you about eating with your brace?	***	***	**
14	Did the advice of your dentist make you change the foods you eat?	***	***	NS
15	How often do you avoid eating foods if you are unable to brush your teeth/clean brace after meal?	***	***	NS
Total (13 ± 14 ± 15)		***	***	NS
Domain 6: Enjoyment of food				
16	When wearing your brace, can you eat the foods you want to?	***	***	***
17	Do you feel embarrassed when you eat with your brace?	***	***	*
Total (16 ± 17)		***	***	***
Total score		***	***	***

*Note*: An SD indicates standard deviation; T0, baseline data; T1, 1 month after the onset of orthodontic treatment; T2, 3 months after the onset of orthodontic treatment; T3, 6 months after the onset of orthodontic treatment.

Abbreviations: NS, nonsignificant; OHIP‐14, Oral Health Impact Profile 14; ERQoL, eating‐related quality of life.

* *p* < .05;

** *p* < .01;

*** *p* < .001.

Repeated measures analysis of variance with Greenhouse–Geisser correction and pairwise comparisons by the Bonferroni test were used to assess the effect of orthodontic treatment on OHIP‐14 total score based on gender. According to the results, no significant difference existed between males and females in this respect (*p* = .898). The trend of change in total score for both males and females was ascending at first and then descending and significant at T3 and T4 (*p* < .001). Assessment of the effect of orthodontic treatment on ERQoL total score based on gender by using repeated measures analysis of variance with Greenhouse–Geisser correction and pairwise comparisons by the Bonferroni test showed no significant difference in ERQoL total score between males and females (*p* = .191). The change in total score for both males and females was descending and significant over time (*p* < .001).

## 4 DISCUSSION

It has been demonstrated that correction of malocclusion significantly improves the OHRQoL (Andiappan et al., 2015; Javidi et al., 2017). Many studies have reported significant improvement of OHRQoL upon the completion of orthodontic treatment (Andiappan et al., 2015; Jena et al., 2020; Olkun & Sayar, 2019). With respect to the effect of orthodontic treatment on OHRQoL of patients, the trend of change in the present study was such that the OHIP‐14 total score significantly increased at the end of the first month of treatment compared with the preoperative state (indicating a deterioration of the QoL); whereas, at the end of 3 months, this score significantly decreased compared with baseline and 1 month. At 6 months after the treatment onset, the OHIP‐14 total score significantly decreased compared with baseline, 1 month, and 3 months (indicating an improvement in the QoL). Other studies have shown a trend of change relatively similar to that in the present study, and reported a deterioration in the QoL at the onset of orthodontic treatment followed by an improvement over time (Agbaje et al., 2018; Farzanegan et al., 2015; Jena et al., 2020; Johal et al., 2015; Liu et al., 2011). Nonetheless, differences exist between different time points during the course of treatment in which QoL improves or deteriorates. Jena et al. (2020) observed a significant reduction in the QoL at 1 and 3 months after the onset of treatment while they noticed a significant improvement in the QoL at the end of the first year of treatment compared with baseline (preoperative state). Farzanegan et al. (2015) showed a reduction in OHRQoL total score at 2 months after the treatment onset and a significant improvement at 6 months compared with the preoperative state. However, some other studies demonstrated that despite an improvement in the QoL over time, the OHRQoL remained unchanged compared with the baseline (Gao et al., 2021). Furthermore, Johal et al. (2015) reported that the OHRQoL total score decreased in the first 3 months after the treatment onset and then gradually improved at 6 months posttreatment. Agbaje et al. (2018) used the United Kingdom OHRQoL questionnaire and indicated that the OHRQoL of patients significantly decreased during the first week following the treatment onset. They showed a trend of improvement in OHRQoL at 1, 3, and 6 months after the treatment onset; however, this change was not significant compared with the preoperative state, which was in contrast to our findings.

**Figure 1:**
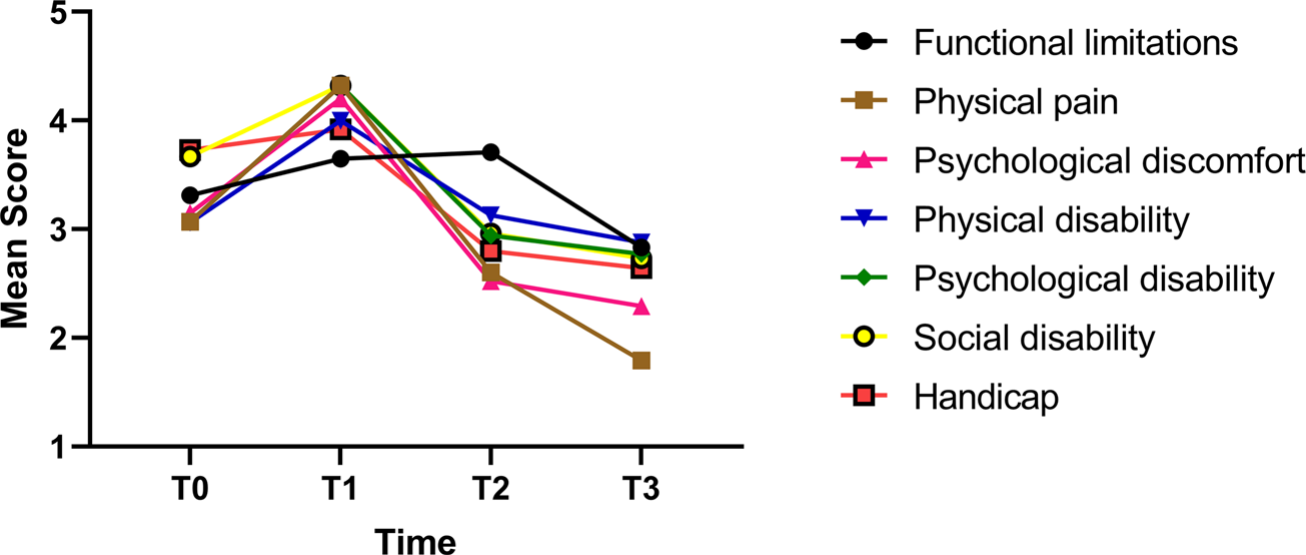
Trend of changes in Oral Health Impact Profile domains at different time points

Based on the current results (Figure 1), the patients had a poorer status in physical pain, psychological discomfort, psychological disability, and social disability domains at the end of the first month compared with the preoperative state. Their status improved at 3 and 6 months and experienced a significant improvement compared with baseline. Similarly, Jena et al. (2020) reported significant deterioration of psychological disability and social disability at 1 month, which later improved. A similar trend was observed for physical disability with the difference that although the status of patients improved at 6 months, the improvement was not significant compared with the preoperative state. With respect to the functional limitation domain, the status of patients significantly deteriorated at 3 months after the treatment onset. At the end of 6 months, functional limitations significantly decreased compared with the prior three time points; this finding was in agreement with the results reported by Farzanegan et al. (2015) Handicap was the only domain that was not negatively affected by orthodontic treatment, and had a better status at 3 and 6 months, compared with baseline. Such a gradual improvement in all domains can be due to improved adaptation to treatment and correction of malocclusion (Jena et al., 2020). The results of studies regarding different domains are highly variable due to the cultural and social status of different populations.

Evidence shows that orthodontic treatment has the greatest impact on eating behavior among the daily activities; however, it is not known which domain is influenced with regard to diet (Moeintaghavi et al., 2013). Thus, since different aspects of eating during orthodontic treatment are not evaluated by the conventional questionnaires, this study used a specific questionnaire designed for this purpose. This questionnaire can provide more information regarding the eating‐related problems; accordingly, the clinicians can provide patients with more effective instructions regarding diet during the course of treatment. The current results revealed that the trend of change in ERQoL total score was descending during the course of treatment, and significant differences existed in this respect between all three time points. It means that the factors related to diet of orthodontic patients improved at 3 and 6 months compared with 1 month. Similarly, Choi et al. (2017) found that patients under orthodontic treatment have problems in eating and limitations in selection of foods. In their qualitative study, they observed that patients experienced an improvement in their eating status over time. Nonetheless, they did not report any time points in their study. Also, Abed Al Jawad et al. (2012) reported that the nutritional status improved after a couple of days or weeks following the resolution of initial pain due to installation and activation of appliances. Trein et al. (2013) evaluated the masticatory function and concluded that size of food particles taken returned to normal at 1 month after treatment even in presence of pain in orthodontic patients. This finding was in agreement with our results regarding the improvement in the amount of food taken and the ability to eat a wide variety of foods similar to the pretreatment time. Nonetheless, they only reported results up to 1 month after the treatment onset while the patients were assessed for up to 6 months, posttreatment in our study. The present study revealed a significant improvement in most items individually over time (mainly at 3 months) and this trend continued for up to 6 months; although in some items (related to the first three domains of the questionnaire), despite an improvement over time, the status still remained suboptimal and the patients still complained about them.

In the present study, no significant difference existed between males and females at any time point regarding the effect of orthodontic treatment on OHIP‐14 total score. This result was in line with some previous findings (Sobouti et al., 2020).

Success of orthodontic treatment depends on optimal patient cooperation (Carter et al., 2015). The current results can help dental clinicians to inform the patients regarding the possible consequences of orthodontic treatment and its effect on their QoL, and the temporary deterioration of their QoL and nutrition at specific periods of time. Also, they can ensure the patients that these problems only last for a short period of time after the treatment onset and will resolve over time. By doing so, the patient expectations can be managed and the patient cooperation and compliance with treatment would improve. Moreover, dental clinicians can compile a nutritional instruction for their patients based on the results of the present study.

Several factors such as age, severity of malocclusion, and socioeconomic status of patients can affect the results of such studies (Choi et al., 2016; Dalaie et al., 2018; Nascimento et al., 2016). Choi et al. (2016) found that older patients had a more negative attitude and perception of OHRQoL before treatment. Moreover, it has been demonstrated that aging negatively affects the masticatory function (Choi et al., 2016). Also, the mastication pattern and the consumed food items differ at different ages (Kohyama et al., 2003). The reported results regarding the effect of severity of malocclusion on the OHRQoL and the masticatory function have been controversial (Choi et al., 2016; Dalaie et al., 2018; Johny et al., 2018; Taylor et al., 2009). The main objective of the present study was to assess the OHRQoL and ERQoL of orthodontic patients. After data collection and analysis, the results indicated the homogeneity of the patients with regard to the confounding variables such as age, severity of malocclusion, and socioeconomic status, such that a high percentage (above 90%) of the participants were between 23 and 29 years. Also, the majority of participants (95%) were selected among those presenting to dental school clinic (which has a low fee for service), and therefore, had almost the same socioeconomic status. Furthermore, most participants (81.9%) were classified in Grade III of IOTN‐DHC. Thus, it may be concluded that the abovementioned confounders could not affect the results. However, this fact decreases the generalizability of the present results to the entire population of orthodontic patients (different ages, different severities of malocclusion, and different socioeconomic levels). Nonetheless, some other confounders are still present that could have affected the results, which was a limitation of this study. For instance, esthetics is an IOTN component, which was not addressed in this study. Another confounding factor, which should have been taken into account, is the level of education of patients, which can affect the perception of patients from their OHRQoL (Heravi et al., 2011). The diet of patients is another factor to consider, although patients with special diets were excluded from this study. The participants of the present study had a comparable socioeconomic level and a probably comparable diet. However, further studies with a larger sample size and longer follow‐ups are required to address the limitations of the present study. Also, the effect of type of fixed orthodontic appliance and the orthodontic bracket type should be investigated in future studies. The latter parameter could not be compared in this study with adequate statistical power.

## 5 CONCLUSION

Within the limitations of this study, it may be concluded that the OHRQoL is adversely, but temporarily, affected by the onset of orthodontic treatment. Nonetheless, this trend is expected to improve within 3 months following the treatment onset. The eating problems caused by orthodontic treatment also resolve with time, resulting in an improvement in ERQoL. Enhanced knowledge of clinicians about this topic and raising the awareness of orthodontic patients regarding the possible problems during the course of treatment and their gradual resolution can greatly improve the success of treatment. Generalization of the current results must be done with caution, and future studies with a larger sample size and longer follow‐ups are required on the effects of possible influential parameters such as age, severity of malocclusion, socioeconomic status, and level of education of patients.

## AUTHOR CONTRIBUTIONS

**Yasamin Babaee Hemmati**: Conceptualization; methodology; approving final version of manuscript. **Arastoo Mirmoyed**: Acquisition of data; investigation. **Mohammad Ebrahim Ghaffari**: Analysis and interpretation of data; software. **Mehran Falahchai**: Writing—original draft; supervision.

## CONFLICT OF INTEREST

The authors declare no conflict of interest.

## Data Availability

The data that support the findings of this study are available from the corresponding author upon reasonable request.
